# Hepatic and Aortic Arch Expression and Serum Levels of Syndecan-1 in ApoE^-/-^ Mice

**DOI:** 10.2174/1874091X01711010077

**Published:** 2017-09-21

**Authors:** Elena I. Leonova, Elena S. Sadovnikova, Elvira R. Shaykhutdinova, Oxana V. Galzitskaya, Arkady N. Murashev, Alexandr S. Solonin

**Affiliations:** 1 Institute of Biochemistry and Physiology of Microorganisms, Russian Academy of Sciences, Moscow Region, Pushchino, 142290, Russia; 2 Branch of Shemyakin-Ovchinnikov Institute of Bioorganic Chemistry, Russian Academy of Sciences, Pushchino 142290, Russia; 3 Institute of Protein Research, Russian Academy of Sciences, Moscow Region, Pushchino, Russia

**Keywords:** Syndecan-1, ApoE^-/-^ mice, C57Black mice, Disordered structure, Cholesterol level, HSPG

## Abstract

**Background:**

Heparan sulfate proteoglycan (HSPG) syndecan-1 (Sdc1) acts as a receptor for triglyceride-rich lipoproteins (TRLs), growth factors, chemokines and enzymes. Due to the disordered structure, its function is as diverse as its ligands. In this paper, we have analyzed hepatic and aortic arch expression of Sdc1 in ApoE^-/-^ mice and examined their association with biochemical changes in plasma during the atheroma formation.

**Methods:**

ApoE knockout (ApoE^-/-^) mice as a model of atherosclerosis were used. Plasma chemistry parameters were estimated by automatic biochemical analyzer. The ELISA test was used to detect soluble Sdc1. The mRNA level of syndecan-1 in liver cells and aortic arch was determined by real time PCR.

**Results:**

The Sdc1 mRNA level in liver cells was 1.5-2.5 times higher in ApoE^-/-^ mice compared to the wild-type species and increased with age, whereas it remained at the same level in wild-type mice upon aging. Furthermore, the plasma cholesterol level was 4-6 times higher in ApoE^-/-^ mice compared to the wild type; in contrast, triglyceride (TG) remained at the same level. Simultaneously, the expression of Sdc1 in the aortic arch of ApoE^-/-^ mice decreases with age; however, it increases in wild-type mice of the same age. We determined that the Sdc1 mRNA expression in liver cells is significantly higher compared to the cells of aortic arch. In addition, our research demonstrated that the level of soluble Sdc1 slightly increased with age and did not depend on mouse genotype; yet, the total amount of soluble Sdc1 was higher in ApoE^-/-^ mice.

**Conclusion:**

Our data suggest that the level of soluble Sdc1 in serum of mice can be associated with chronic inflammation. In addition, we hypothesized that a compensatory increase in the Sdc1 expression in ApoE^-/-^ mice may prevent accumulation of triglycerides in serum, yet having no effect on cholesterol accumulation.

## 1 INTRODUCTION

**Fig. (1):**
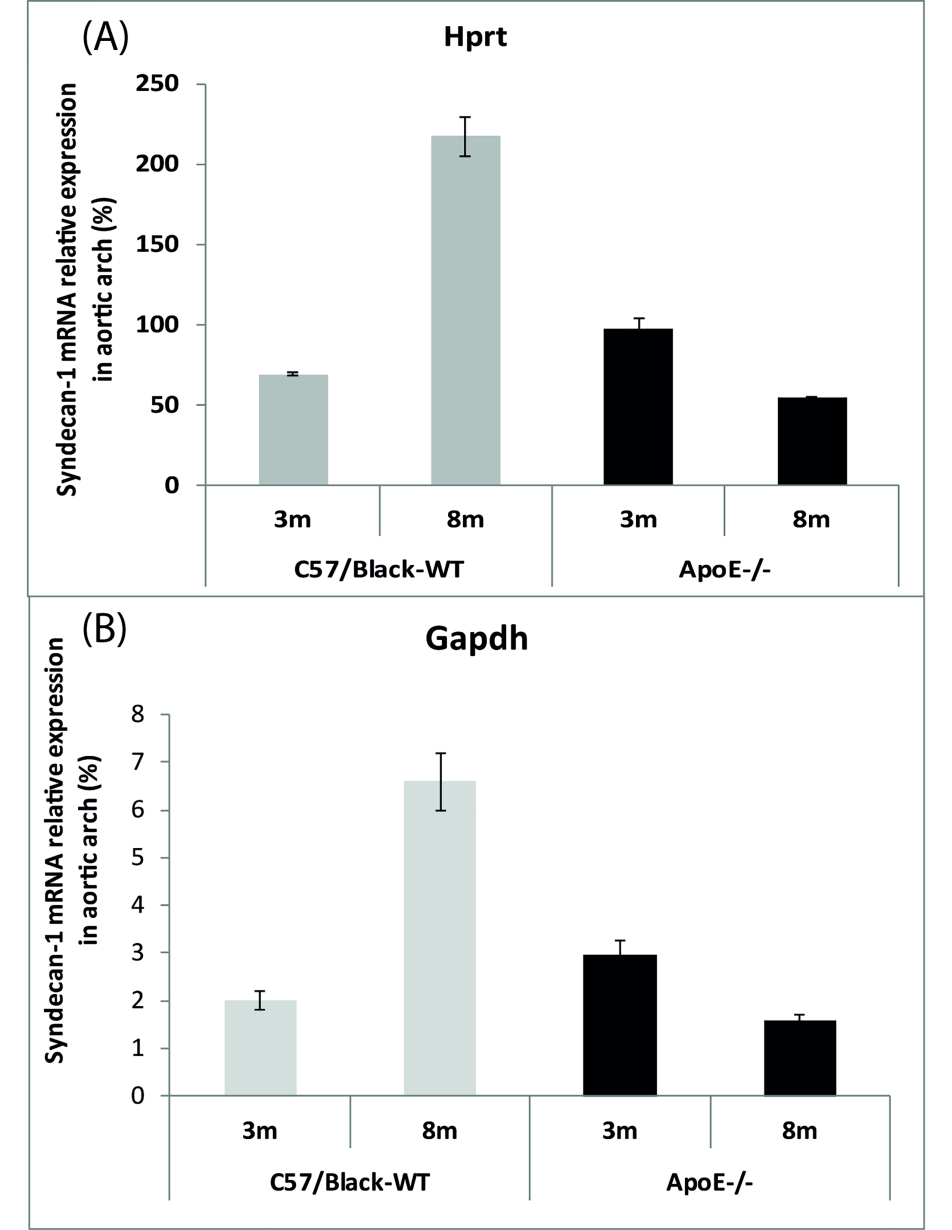
Quantitative syndecan1 mRNA analysis in aortic arch of ApoE^-/-^ mice and C57 Black mice. A: htrp represents relative gene expression of Sdc1 (*n* = 6, *p* < 0.05). B: The graph represents relative gene expression of Sdc1 (*n* = 6, *p* < 0.05).

Sdc1 is HSPG found on the surface of different types of mammalian cells. It belongs to a four-member family of cell surface HSPGs [1]. These molecules are categorized based on their structure and localization. Sdc1 contains an *N*-terminal signal peptide, an ectodomain, a transmembrane domain, and a short *C*-terminal cytoplasmic domain. The ectodomain of Sdc1 is covalently attached by heparan sulfate and chondroitin sulfate chains [2]. Sdc1 can function as a receptor and co-receptor modulating the primary signaling receptors at the cell surface interacting with a large number of potential ligands [1]. As a consequence, Sdc1 participates in diverse cellular activities including cell-cell adhesion [3], wound healing, extracellular matrix organization [4], binding growth factors [5] and clearance of triacylglycerol-rich lipoproteins (TRLs) [6, 7]. It may be suggested that the plasticity of the disordered ectodomain of Sdc1 determines its capacity to respond quickly to environmental changes [8]. As a component of the endothelial glycocalyx (EG), syndecan-1 plays a crucial role in regulation of vascular permeability, prevention of blood cell migration to the vessel wall, and transmission of shear stress [9]. It is known that the expression of Sdc1 is highly regulated and depends on the tissue type and the developmental stage [2]. Sdc1 is actively secreted by macrophages and can be enhanced by angiotensin-2 [10]. Gene expression of Sdc1 in B lymphocytes is activated just after their differentiation into antibody-producing plasma cells. For this reason, Sdc1 is widely used as a biomarker of plasma cells [11, 12]. Sdc1 is actively synthesized by hepatocytes, where it also serves as an additional receptor of TRLs [7]. Interestingly, mouse hepatocytes express syndecan-1, -2, and -4, yet the only Sdc1 is the primary hepatocyte HSPG receptor mediating the clearance of TRLs [13]. ApoE acts as a receptor-binding ligand at the surface of chylomicrons and VLDL (very low density lipoproteins) [14] for receptors in the liver, which include the low density lipoprotein receptor (Ldlr), low density lipoprotein-related protein-1 (Lrp1), and HSPG Sdc1 [15, 16]. As ApoE is the main ligand that mediates TRLs binding and clearance by many types of receptors, deletion of this gene causes broken fat metabolism in mice (Fig. **1**). As a result, six-month-old ApoE^-/-^ mice form atherosclerotic plaques even on a normal diet [17]. Consequently, ApoE^-/-^ mice provided the first practical model of hyperlipidemia and atherosclerosis. Sdc1 can also mediate the clearance of TRLs through the ApoAV ligand, which enables its action in the absence of the ApoE ligand [13, 18-20].

There are two forms of Sdc1: incorporated into the membrane and soluble, when the ectodomain can be shed from the membrane surface. This process is mediated by extracellular zinc-depended endopeptidases and metalloproteinases [21]. Soluble Sdc1, on one hand, accelerates migration of leukocytes into the intima, thus promoting inflammation [22, 23]. On the other hand, any kind of inflammation can enhance shedding of Sdc1 resulting in its accumulation in the interstitial fluid around the wounds [24]. Nonetheless, the exact mechanism of shedding activation has not been fully determined yet. In healthy organisms, the concentration of soluble Sdc1 is quite low [25]. A high level of soluble Sdc1 is very typical of the tissue recovering after damage or in response to inflammation [26]. In addition, intensive shedding of Sdc1 from the surface of hepatocytes may cause hypertriglyceridemia [27].

We set out to determine how the Sdc1 aortic arch, hepatic expression of Sdc1 in ApoE^-/-^ mice is associated with biochemical changes in plasma during the atheroma formation.

## 2 MATERIALS AND METHODS

Our experimental mice were kept under standard conditions. The study was done in accordance with the standards of care and ethical treatment of animals admitted by the European Convention for the Protection of Vertebrate Animals Used for Research in 1986 in Strasbourg. Experimental mice were obtained from the Branch of the Shemyakin–Ovchinnikov Institute of Bioorganic Chemistry, Russian Academy of Sciences. They were divided into four groups according to genotype and age (Table **1**).

### 2.1 Isolation of RNA

For taking tissue samples, mice were euthanized in a CO_2_ chamber. The aortic arch was cut out under a binocular microscope, and liver samples not exceeding 20 μg were taken. All samples were immediately frozen in liquid nitrogen. Frozen tissue samples were stored at -70°C.

Total RNA was isolated using the trizol method. The aortic arch and the liver sample were homogenized for 3 min at the frequency of 50 Hz using a TissueLyser machine in the presence of 0.5 ml trizol (Ambion). The homogenate was incubated for 5min at room temperature to complete dissociation of protein–nucleic acid complexes. 100 μl of chloroform was added. The mixture was shaken vigorously for 15s and incubated for 3min at room temperature. The homogenate was centrifuged at +4°C, 15min at 12000 *g*. The supernatant was collected and transferred to a sterile tube. To precipitate the RNA, an equal volume of isopropanol was added; the mixture was incubated for 10min at room temperature and then centrifuged at +4°C for 10min at 12000 *g*. The RNA pellet was washed twice by adding 500 μl of 75% ethanol, air-dried and finally dissolved in 30 μl of deionized water. The RNA concentration was measured using a NanoDrop spectrophotometer. Before the additional purification with an RNease minikit (Qiagen) the isolated total RNA was treated with DNase (Fermentas) (Supplementary Material, Table **1**). Finally, the RNA concentration was measured using a NanoDrop spectrophotometer and the preparation was frozen at -70°C. The quality of total RNA was evaluated by the ratio260:280. The ratio of A260/A280 ≥ 2.0 shows a high quality of the RNA.

### 2.2 Synthesis of the First Strand cDNA

For the first strand cDNA synthesis we mixed 20 pM of oligo d(T)20 primer, 0.3 mg of the total isolated RNA and deionized water. The total volume of 15 μl was incubated at 65°C for 5 min, then in the reaction 4 μl of 5X buffer of reverse transcriptase with 15 mM MgCl_2_, 1 μl of 20 mM dNTPs mix, 0.25 μl of RNase inhibitor (40 u/μl) and 0.1 μl of reverse transcriptase (“Thermo”) (200 u/μl) was added and the mixture was incubated for 30min at 50°C and after that for 5 min at 85°C to stop the reaction. The cDNA was stored at -20°C.

### 2.3 Determination of mRNA Expression of Sdc1 by RTPCR

The amount of cDNA was measured in real time after each amplification cycle. The detection of accumulation of the amplicons was measured by using the SYBRGreen fluorescent intercalating dye. The work was carried out on a DNA-Technology (Russia) thermocycler. Primers for the three reference genes hypoxanthine guanine phosphoribosyl (hprt), glycerol 3-phosphate dehydrogenase, aldehyde (gapdh) and beta-actin (actb) and for the sdc1 gene were used. To select the primers, we used the databases GenBank, MouseGenome, RTPrimerDB, and programs Primer-3plus and Primer 3 web. Synthesis of the primers was performed by Syntol (Russia) (Supplementary Material, Table **2**). The reaction mixture was prepared in a total volume of 25 μl per tube (Supplementary Material, Table **3**). The PCR program was as follows: 3 min melting at 94°C, then 40 cycles (10s at 94°C and 45s at 60°C). To construct the melting curve, the PCR program was continued by a gradual melting temperature to denature the DNA up to 95°C.

Analysis was performed by direct comparison of DNA accumulation graphs of the Cp values of the quantity of second derivatives. The comparison of mRNA of the Sdc1 expression was performed relative to the reference genes [28]. The maximum values of the second derivatives are more accurate due to their location on the exponential growth curve. The average Cp value was automatically determined by a detecting device (a thermocycler). The effectiveness was estimated by the equation: Е = 10^(-1/а)^, where a is the difference in the Cp values determined by a 10-fold dilution of the sample. The mRNA expression level of Sdc1 (X) relative to the reference gene (Y) is determined by the equation: [Х]/[У] = E_у_^СрУ^/E_Х_^СрХ^, where СрУ and СрХ are the values of the threshold cycles of Ср.

### 2.4 ELISA Measurements of Soluble Syndecan-1 Concentration

The concentration of soluble syndecan-1 in blood serum of experimental mice was determined by the sandwich ELISA (enzyme-linked immunosorbent assay) test. The test is based on antigen-antibody interaction. Blood samples were collected from the orbital venous sinus of mice under general anesthesia. To obtain the serum, the blood samples were kept overnight at +4°C, and then centrifuged for 20min at 3000 rpm. The collected serum was frozen at -70°C. Further, the analyzed serum was added into a 96-well microliter plate with immobilized antibodies specific to Sdc1. To form a solid phase antibody–antigen complex, the plate was incubated for 2h at 37°C. Then, after removing the liquid phase, the biotin-labeled secondary antibodies were added to bind the antigen and another epitope of syndecan-1. At this stage, the antigen immune complex was formed from immobilized molecules and biotinylated antibodies, which resembled a “sandwich” (hence the name of the method). The secondary incubation lasted for 1h at 37°C, after which the plate was washed three times to remove the conjugate by a special washing buffer. Then, a peroxidase-labeled avidin conjugate was added. Avidin has four high affinity biotin-binding sites. This incubation lasted for 30min at 37°C, after which all wells were washed five times. After that the substrate tetramethylbenzidine (TMB) was added to color the peroxidase substrate. The reaction was performed in the dark at 37°C and continued for about 20 min. Finally, to stop the coloring process, a stop reagent was added. The intensity of staining correlated with the number of detected antibodies specific to syndecan-1. The measurements were performed at a wavelength of 450 nm using a spectrophotometer.

The quantitative evaluation was performed using the data from the calibration curve based on standards diluted in several steps, with the already known concentration of antibodies to Sdc1 (Supplementary Material, Table **4**). The final concentration of Sdc1 was multiplied by the ratio of the 20-fold diluted initial serum (Supplementary Material, Fig. **1**).

### 2.5 Serum Chemistry Analysis

Clinical chemistry parameters in serum were measured on a SAPPHIRE 400 (Prestige 24i) automatic biochemical analyzer (Tokyo Boeki, Japan) using reagents from Randox Laboratories Ltd: total protein (the biuret reaction method); albumin (the bromocresol green method); urea (the kinetic method with urease); creatinine (the method with alkaline picrate without deproteinization); cholesterol (the cholesteroloxidase method); triglycerides (the lipase and glycerokinase method); alanine aminotransferase (ALT) and aspartate aminotransferase (AST) (the tris buffer method without pyridoxal-5-phosphate, 37°C); alkaline phosphatase (AP) (the p-nitrophenylphosphate method, optimized IFCC); calcium (the arsenaso method); inorganic phosphates (the UV phosphomolybdate measurement method); chlorides (the colorimetric method). For internal quality control, commercial human control sera (Randox) were used.

### 2.6 Statistical Processing of the Data

Statistical processing of the concentration values of soluble Sdc1 in serum and mRNA expression levels of Sdc1 was performed by the Student's *t-*test which allows evaluating a relatively small data selection. The Student’s distribution has properties of a normal distribution for small data selection and has the same value as for large ones. Score confidence probability *p* is determined by the *t*-test table. There are three levels of statistics significance. The first level of significance (*p* < 0.05) means that the accepted error does not exceed 5%, the second (*p* < 0.01) shows that the error is no more than 1%, and in the third (*p* < 0.001) the error is no more than 0.1%. Data are presented as means ± SD. Statistical analyses were performed with STATISTICA 7.1 software (StatSoft Inc.). Data were analyzed for normality using the Shapiro–Wilk’s test. In the case of normal distribution, the differences between two groups were analyzed with the unpaired two-sided Student’s *t*-test. In the other case, the Mann–Whitney U-test was applied. The 0.05 level of significance was used for statistical evaluation.

## 3 RESULTS

Males from the group of experimental mice (Table **1**) were chosen to measure the mRNA expression of Sdc1 in the aortic arch and liver by RTPCR. Thus, 24 of the aortic arch samples and 24 of the liver tissue samples were analyzed. The analysis was performed by a direct comparison of DNA accumulation graphs (Cp) of Sdc1 to the reference genes.

Only two of the three reference genes were selected. One was the hprt gene and the other the gapdh gene. This choice was based on the fact that in liver cells of wild-type 8-month-old mice the gene expression of actb decreases compared to that of the other genes, which remained on the same level (Supplementary Material, Table **5**). The hprt gene encodes the protein responsible for the exchange of purines in the cell, and the gapdh gene encodes the enzyme, which plays an important role in the process of glycolysis. Their expression is considered to be relatively constant in various types of tissues, and these genes are found to be reliable for the semi-quantitative analysis by RTPCR [28]. Our data showed that the difference in the threshold cycles (ΔCp) of the hprt and gapdh genes was very close in values (Supplementary Material, Table **5**). This makes it possible to analyze the changes in gene expression of Sdc1 in samples of experimental animals (Supplementary Material, Tables **6**-**9**). Statistical processing of the data by the Student's *t*-test revealed significant differences between experimental groups (Supplementary Material, Tables **10**, **11**).

Based on these results, it was found that the percent size of mRNA of Sdc1 in the walls of the aortic arch dramatically increases with age (*p* <0,001) in wild-type mice and is reduced (*p* < 0.05) in ApoE^-/-^mice (Fig. **1**).

**Fig. (2):**
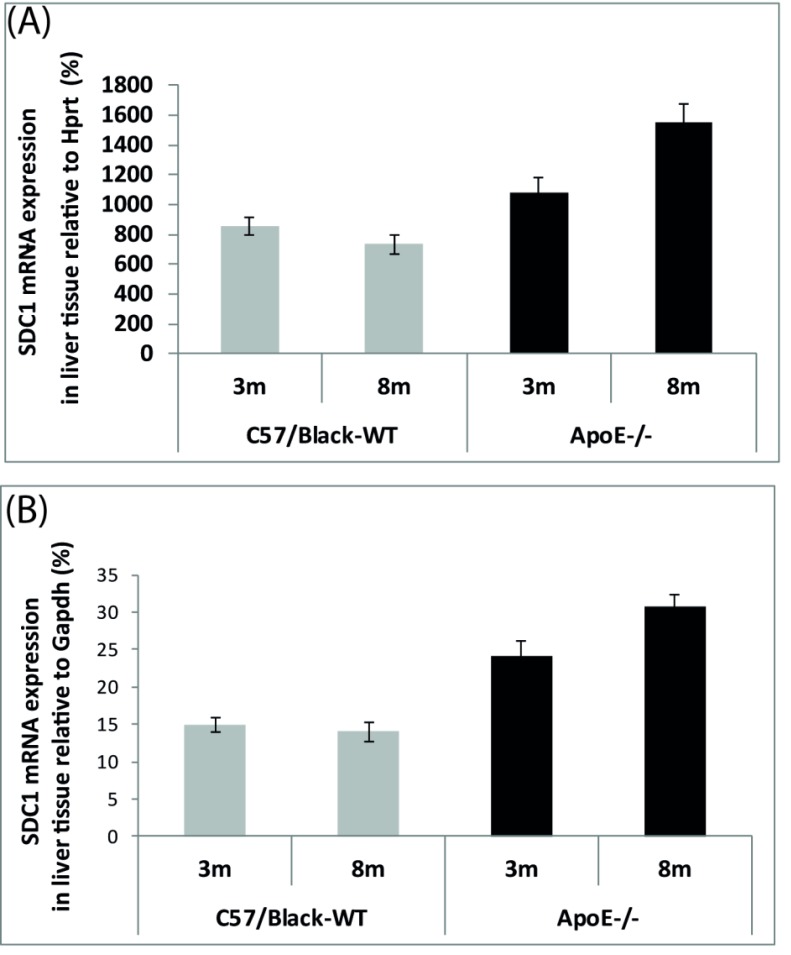
Quantitative syndecan1 mRNA analysis in the liver of ApoE^-/-^mice and C57Black mice. A: htrp represents relative gene expression of Sdc1 (*n* = 6, *p* < 0.05). B: The graph represents relative gene expression of Sdc1 (*n* = 6, *p* < 0.05).

Analysis of the liver tissue showed that the relative mRNA levels of mRNA Sdc1 increased in ApoE^-/-^mice with age (*p* <0.001) (Fig. **2**) and did not change in wild-type mice (*p* < 0.05).

It was shown that the relative mRNA levels of Sdc1 in the liver cells are much higher compared to the aortic arch cells of experimental mice.

**Fig. (3):**
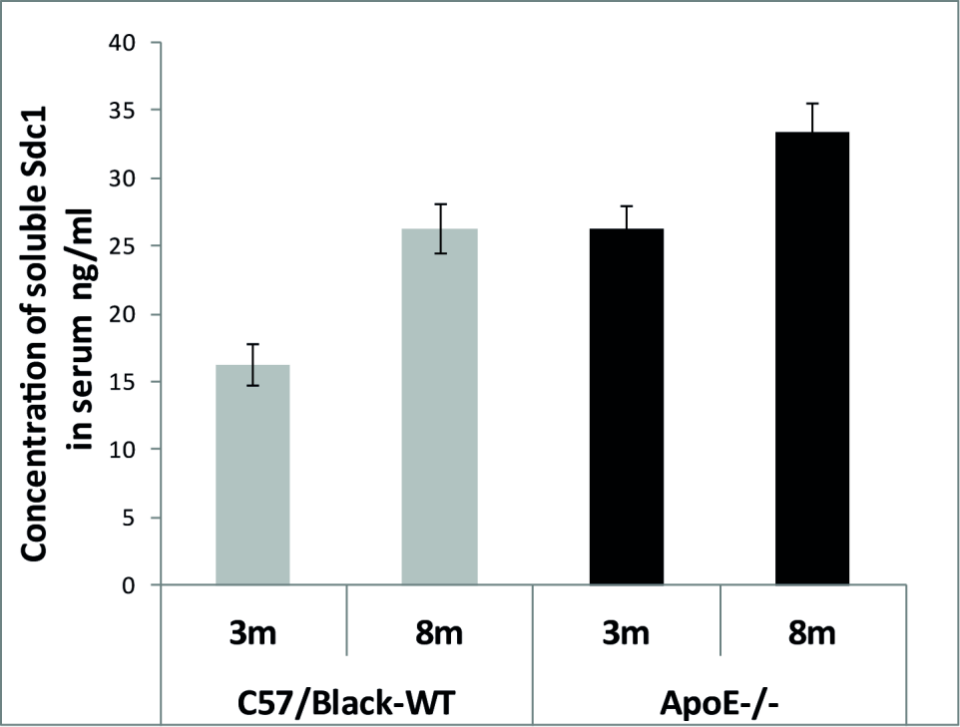
Levels of soluble Sdc1 in blood serum of experimental mice estimated using the enzyme immunoassay.

The concentration of soluble Sdc1 in blood serum of experimental mice was determined using the ELISA method. The blood serum was collected from the orbital venous sinus under general anesthesia of experimental animals (Table **1**). Hence 48 blood samples were examined and each trial was performed twice (Supplementary Material, Tables **12**-**15**). The Student’s *t*-test revealed great differences in the statistics significance between groups (*n* = 12, *p* < 0.001) (Supplementary Material, Tables **16**, **17**). The results indicate that the level of soluble Sdc1 in serum increases with age regardless of the genotype, but in general it is higher in the ApoE^-/-^mice (Fig. **3**).

Data on the serum biochemistry parameters in ApoE^-/-^ and C57Black mice are given in Table **2**. Our results show that deletion of the АpoE gene in mice results in a 4–6 times higher cholesterol level than in С57Black mice (Table **2**). In contrast, the triglyceride level and other parameters are only negligibly affected and remain within the normal limits established for C57Black mice (**Charles River Laboratories**) [29].

## 4 DISCUSSION

In our experiments the control C57Black mice were prone to obesity. We noticed that 8-month-old wild-type mice were much fatter compared to ApoE^-/-^mice of the same age. For this reason, C57Black mice were used to generate ApoE^-/-^ mice. The first mouse carrying the ApoE knockout gene was created by Nobuyo Maeda in 1991 [30]. Along with it, she tried to get mice with hypercholesterolemia by destroying the ApoB100 and ApoA1 genes, but neither of mutants showed atheroma formation. Exactly the opposite, apoB100 knockout mice had decreased levels of LDL particles [31, 32], and ApoA1 knockout mice had reduced plasma cholesterol levels [33]. Finally, the lack of ApoE caused severe hypercholesterolemia in mice. Nobuyo Maeda suggested a possible explanation of the high cholesterol level as significant accumulation of TRLs particles in mouse plasma [30]. And it was quite logical, because ApoE is the main ligand for hepatic TRLs receptors, including Sdc1 [13, 15, 16, 18-20]. Because the plasma TG level correlates directly with the plasma TRLs level, we measured the TG level [34]. Yet, our result showed that the plasma TG level remained within the normal range and did not depend on the genotype (Charles River Laboratories) [29]. The same result was obtained in 1996 by the Havekes group [35]. Even the claim by Mohammed H. Moghadasian & Сo that ApoE^-/-^ mice have a two times higher level of plasma TG compared to the wild type [36] demonstrated that the concentration of TG in all groups of mice was within the normal range, according to the data of Charles River Laboratories and of Fernandez & Сo group [29]. It might be possible that destruction of one gene can activate compensatory mechanisms to offset the “knockout”. It is known that, besides the ApoE ligand that is crucial for function of Ldlr and Lrp1 receptors, Sdc1 can mediate the hepatic clearance of TRLs through the ApoA ligand [7]. In addition, the Havekes group have found a dramatically high TG level in the liver (+232%) tissue of ApoE^-/-^ mice [35], which also supports our idea of TRLs clearance by hepatic Sdc1. Further research is required to determine whether or not a compensatory increase in hepatic Sdc1 expression can prevent the hypertriglyceridemia in ApoE^-/-^ mice. In addition, we hypothesize that the high cholesterol level in ApoE^-/-^ mice can be a result of interruption of reverse transport of cholesterol. It has been shown that elimination of ApoE leads to significant disruption of HDL. As a consequence, cells stop synthesizing new LDL receptors in response to cholesterol supply and at the same time LDL accumulate in serum.

We observed that all 8-month-old ApoE^-/-^mice had atherosclerotic plaques in the aorta, which could be easily visualized with a binocular microscope. In contrast, there were no atherosclerotic plaques in the aorta of 8-month-old C57Black mice, although they were very fat. It should be emphasized that the syndecan-1 gene in the aortic arch was expressed at a very low level in all groups of mice. At the same time, we want to bring to notice that it increased in C57Black mice and decreased in ApoE^-/-^ mice with age. We suggest that the increasing level of the Sdc1 gene expression in the aortic arch of C57Black mice might contribute to keeping EG of vessels safe and healthy. At the same time, the decreasing level of the Sdc1 gene expression in the aortic arch of ApoE^-/-^ mice supports the idea that the high LDL level in serum leads to cholesterol accumulation in the arterial wall. Such cholesterol invasion in the vessel’s wall leads to endothelium damage and atheroma formation.

In addition, our results have shown that the level of soluble syndecan-1 increases slightly with age and does not depend on mouse genotype. In any case, the total amount of soluble syndecan-1 is higher in ApoE^-/-^ mice. Оur findings indicate that further studies should be conducted to evaluate the effect of Sdc1 on the lipid metabolism, as well as on the long-term safety of vessel-wall EG.

## Figures and Tables

**Supplement-Fig. (1):**
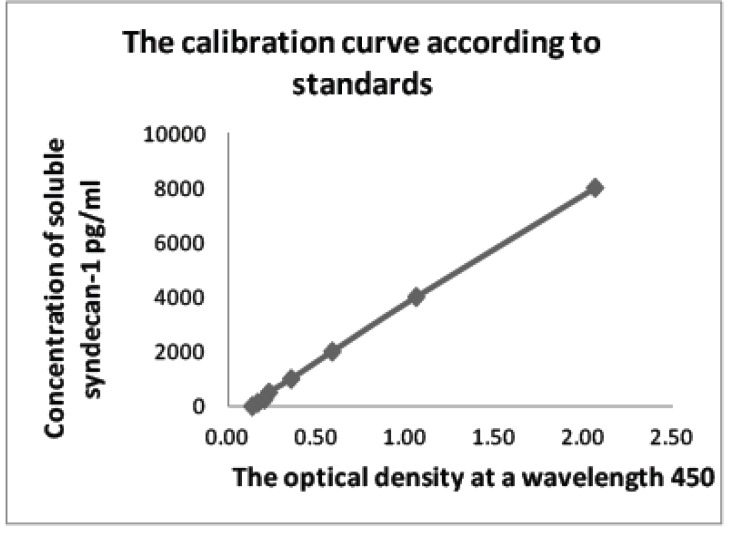
Calibration Curve based on standards diluted in several steps.

**Table 1:** Experimental groups of mice.

Group #	Genotype	Age	Quantity	Mass (g)
		(months)		
1	АроЕ^-/-^	3	12 males	25.6 ±1.3
2	АроЕ^-/-^	8	12 males	29.7 ± 1.6
3	С57Black	3	12 males	27.1 ± 1.6
4	С57Black	8	12 males	32.2 ± 1.7

**Supplement-Table 1:** Dnase-1 mix for samples.

		V= 30 μL
1.	10Хbuffer (for Taq- polymerase, with 25 mM MgCl_2_and 1mM CaCl_2_)	3 μL
2.	RNA	23.3 μL (1μg)
3.	1u/μL DNAse-1 (RNase free)	3 μL
4.	40 u/ μL RNasin	0.7 μL

**Supplement-Table 2:** Primer sequences.

Gene	Primer Sequences
Hprt1	Hprt1_F: 5’-AGCTACTGTAATGATCAGTCAACG -3’ Hprt1_R: 5’- AGAGGTCCTTTTCACCAGCA -3’
Gapdh	Gapdh _F: 5’- CTCCCACTCTTCCACCTTCG -3’ Gapdh _R: 5’- CCACCACCCTGTTGCTGTAG -3’
Actb	Actb _F: 5’- CAACGAGCGGTTCCGATG – 3’ Actb _R: 5’- GCCACAGGATTCCATACCCA- 3’
Sdc1	Sdc1-F: 5' - GGGCTCTGGAGAACAAGACTTC - 3' Sdc1-R: 5' - CTCCGGCAATGACACCTCC -3'

**Supplement-Table 3:** Real-time PCR mix for samples.

		V= 25μL
1.	10Хbuffer (for Taq- polymerase with SybrGreen))	2.5 μL
2.	2.5 mM dNTPs	2.5 μL
3.	25 mM MgCl_2_	2.5 μL
4.	10 μM Primers	per1.0 μL
6.	cDNA	5 μL
7.	Н_2_О_Q_	10 μL
8.	5000 u/ml Taq_ polymerase	0.25 μL

**Supplement-Table 4:** Optical density values of standards with known concentrations of syndecan-1.

Optical Density 450 nm	Concentration of Syndecan-1, pg/ml
2.06	8000
1.05	4000
0.58	2000
0.35	1000
0.23	500
0.20	250
0.16	125
0.13	0

**Supplement-Table 5:** Values of ΔCp for the reference genes in aortic arch and liver cells.

Liver Tissue	ΔCpHprt_GAPDH	ΔCpGapdh_βact	ΔCpHprt_βact
3 months C57BL-WT	5.8	-1.7	4.1
8 months C57BL-WT	5.7	0.7	6.4
3 months АроЕ-/-	5.5	-1.2	4.3
8 months АроЕ-/-	5.6	-1.2	4.5
Aortic arch	ΔCpHprt_GAPDH
3 months C57BL-WT	5.1
8 months C57BL-WT	5.1
3 months АроЕ-/-	5.1
8 months АроЕ-/-	5.1

**Supplement-Table 6:** Measured data of sdc1 mRNA of 3-month-old male ApoE-/- mice.

Nº of Animal -Aortic Arch	Cp Syndecan-1	Average Value of Ср for Syndecan-1	Cp Hrtp	Average Value of Ср for Hrtp	Ср GAPDH	Average Value of Ср for GAPDH	ΔCp Syn_Hrtp	ΔCp Hprt_GAPDH	ΔCp Syn_GAPDH
1	21.5	21.6	21.9	21.8	16.7	16.7	-0.2	5.1	4.9
	21.6		21.7		16.7				
2	22.7	22.7	22.5	22.5	17.6	17.6	0.1	5.0	5.1
	22.6		22.5		17.5				
3	21.7	21.6	21.6	21.6	16.8	16.8	-0.1	4.8	4.8
	21.4		21.6		16.8				
4	21.5	21.5	21.3	21.3	15.9	16.0	0.3	5.3	5.6
	21.5		21.2		16.0				
5	22.8	22.9	22.6	22.6	17.4	17.4	0.3	5.3	5.5
	22.9		22.6		17.3				
6	22.6	22.7	22.6	22.8	17.9	17.8	-0.1	5.0	4.9
	22.7		22.9		17.6				
		22.1		22.1		17.0	0.04	5.1	5.1
Standard deviation (σ)		0.7		0.6		0.7			
Nº of animal -liver tissue	Cp syndecan-1	Average value of Ср for syndecan-1	Cp Hrtp	Average value of Ср for Hrtp	Ср GAPDH	Average value of Ср for GAPDH	ΔCp Syn_Hrtp	ΔCp Hprt_GAPDH	ΔCp Syn_GAPDH
1	17.9	17.8	20.7	20.8	15.2	15.3	-3.0	5.5	2.5
	17.6		20.8		15.3				
2	16.3	16.3	19.6	19.7	14.3	14.5	-3.4	5.2	1.8
	16.2		19.7		14.6				
3	18.4	18.5	21.7	22.3	16.5	16.5	-3.9	5.8	2.0
	18.5		22.9		16.5				
4	15.3	15.3	18.4	18.6	13.2	13.3	-3.4	5.4	2.0
	15.2		18.8		13.3				
5	15.2	15.3	18.3	18.3	12.9	12.9	-3.0	5.4	2.4
	15.4		18.3		12.9				
6	16.6	16.5	20.2	20.2	14.7	14.7	-3.7	5.5	1.8
	16.3		20.1		14.6				
Average value		16.6		20.0	14.6	14.5	-3.4	5.5	2.1
					14.6				
Standard deviation (σ)		1.3		1.5		1.3			

**Supplement-Table 7:** Measured data of sdc1 mRNA of 8-month-old male WTC57Black mice.

Nº of Animal -Aortic Arch	Cp sdc1	Average Value Ср Sdc1	Cp Hrtp	Average Value Ср Hprt	Cp βact	Average Value Ср βact	Ср GAPDH	Average Value GAPDH	ΔCp Syn_Hprt	ΔCp Syn_βact	ΔCp Hprt_βact	ΔCp Gapdh_βact	ΔCp Hprt_GAPDH	ΔCp Syn_GAPDH
13	22.9	22.6	23.6	23.7	15.4	15.6	18.4	18.5	-1.1	7.0	8.1	2.9	5.2	4.1
	22.2		23.7		15.7		18.5							
14	22.4	22.4	24.2	23.9	17.4	17.5	18.4	18.8	-1.5	5.0	6.4	1.4	5.1	3.6
	22.4		23.5		17.5		19.2							
15	21.9	21.9	22.8	22.8	15.6	15.4	17.7	17.5	-0.9	6.5	7.4	2.1	5.3	4.4
	21.8		22.7		15.1		17.2							
16	22.4	22.3	23.3	23.4	15.7	15.7	18.5	18.6	-1.1	6.6	7.7	2.9	4.8	3.8
	22.2		23.4		15.7		18.6							
17	22.5	22.4	23.5	23.6	16.6	16.7	18.2	18.2	-1.2	5.8	6.9	1.6	5.4	4.2
	22.3		23.6		16.7		18.2							
18	22.9	22.6	23.5	23.6	16.1	16.4	19.2	19.0	-1.0	6.2	7.2	2.6	4.6	3.6
	22.2		23.6		16.7		18.7							
Average Value:		22.3		23.5		16.2		18.4	-1.1	6.2	7.3		5.1	3.9
	
Standard deviation (σ)		0.3		0.4				0.5						
Nº of Animal -Liver Tissue	Cp Sdc1	Average Value Ср Sdc1	Cp Hrtp	Average Value Ср Hprt	Cp βact	Average Value Ср βact	Ср GAPDH	Average Value Ср GAPDH	ΔCp Syn_Hprt	ΔCp Syn_βact	ΔCp Hprt_βact	ΔCp Gapdh_βact	ΔCp Hprt_GAPDH	ΔCp Syn_GAPDH
13	20.5	20.6	23.2	23.2	17.1	17.1	17.9	17.9	-2.7	3.5	6.2	0.8	5.3	2.7
	20.6		23.2		17		17.9							
14	20.2	20.2	23.5	23.4	17.2	17.2	17.7	17.7	-3.2	3.0	6.2	0.6	5.7	2.5
	20.1		23.2		17.1		17.7							
15	19.4	19.3	22.4	22.5	16.7	16.0	16.2	16.2	-3.2	3.3	6.5	0.1	6.3	3.2
	19.2		22.5		15.3		16.1							
16	20.3	20.3	23.2	23.2	16.6	16.4	17.1	17.0	-2.9	3.9	6.8	0.6	6.3	3.4
	20.3		23.2		16.2		16.8							
17	20.5	20.2	22.5	23.0	16.3	16.3	17.3	17.5	-2.8	4.0	6.7	1.2	5.5	2.8
	19.9		23.4		16.2		17.6							
18	19.4	19.5	21.9	21.9	15.9	15.6	16.6	16.7	-2.4	4.0	6.4	1.1	5.3	2.9
	19.6		21.9		15.2		16.7							
Average value:		20.0		22.8		16.4		17.1	-2.8	3.6	6.4	0.7	5.7	2.8
Standard deviation (σ)		0.5		0.6				0.7						

**Supplement-Table 8:** Measured data of sdc1 mRNA of 3-month-old male WTC57Black mice.

Nº of Animal -Aortic Arch	Cp Sdc1	Average Value. Ср Sdc1	Cp Hrtp	Average Value. Ср Hrtp	Ср GAPDH	Average Value. Ср GAPDH	ΔCp Syn_Hrtp	ΔCp Syn_GAPDH	ΔCp 3_up_GAPDH
25	21.1	21.2	20.6	20.7	15.5	15.5	0.5	5.2	5.7
	21.2		20.7		15.5				
26	21.2	21.2	20.6	20.6	15.9	16.0	0.6	4.7	5.3
	21.2		20.6		16.0				
27	21.6	21.6	20.5	21.2	15.7	15.7	0.5	5.5	5.9
	21.6		21.8		15.7				
28	21.1	21.2	20.6	20.7	15.5	15.5	0.5	5.2	5.7
	21.2		20.7		15.5				
29	21.2	21.2	20.6	20.6	15.9	16.0	0.6	4.7	5.3
	21.2		20.6		16.0				
30	21.6	21.6	20.5	21.2	15.7	15.7	0.5	5.5	5.9
	21.6		21.8		15.7				
Average value:		21.4		20.8		15.7	0.5	5.1	5.6

Standard deviation (σ)		0.2		0.3		0.2			
Nº of animal -liver tissue	Cp sdc1	Average value. Ср sdc1	Cp Hrtp	Average value. Ср Hrtp	Ср GAPDH	Average value. Ср GAPDH	ΔCp Syn_Hrtp	ΔCp Syn_GAPDH	ΔCp 3_up_GAPDH
25	22.9	22.6	25.6	25.5	19.6	19.6	-3.0	5.9	3.0
	22.2		25.4		19.5				
26	22	21.8	25.2	25.2	18.9	19.0	-3.4	6.2	2.8
	21.6		25.2		19.1				
27	21.2	20.8	23.7	23.7	18.0	18.0	-2.9	5.7	2.8
	20.4		23.7		17.9				
28	17.9	18.4	21.1	21.2	15.4	15.4	-2.8	5.8	3.0
	18.8		21.2		15.4				
29	19.7	20.0	23.2	23.4	17.3	17.3	-3.4	6.1	2.7
	20.3		23.5		17.3				
30	19.7	19.7	22.6	22.6	17.3	17.3	-3.0	5.3	2.4
	19.6		22.6		17.2				
Average value:		20.5		23.6		17.8	-3.1	5.8	2.8
Standard deviation (σ)		1.5		1.6		1.5			

**Supplement-Table 9:** Measured data of sdc1 mRNA of 8-month-old male ApoE-/-mice.

Nº of Animal -Aortic Arch	Cp Sdc1	Average Value. Ср Sdc1	Cp Hrtp	Average Value. Ср Hprt			Ср GAPDH	Average Value GAPDH	ΔCp Syn_Hprt				ΔCp Hprt_GAPDH		ΔCp Syn_GAPDH
37	20.2	20.2	19.3	19.4	14.7	14.6	0.8	4.9		5.7
	20.2		19.5		14.4					
38	21.4	21.4	20.4	20.5	15.4	15.4	0.9	5.1		6.1
	21.4		20.5		15.3					
39	21.4	21.3	20.4	20.4	15.2	15.2	0.9	5.2		6.1
	21.1		20.4		15.2					
40	20.2	20.2	19.3	19.4	14.7	14.6	0.8	4.9		5.7
	20.2		19.5		14.4					
41	21.4	21.4	20.4	20.5	15.4	15.4	0.9	5.1		6.1
	21.4		20.5		15.3					
42	21.4	21.3	20.4	20.4	15.2	15.2	0.9	5.2		6.1
	21.1		20.4		15.2					
Average value:		20.8		19.9		15.0	0.9	5.1		5.9
Standard deviation (σ)		0.6		0.5				0.4						
Nº of Animal -Liver Tissue	Cp sdc1	Average Value Ср Sdc1	Cp Hrtp	Average Value Ср Hprt	Cp βact	Average Value Ср βact	Ср GAPDH	Average Value Ср GAPDH	ΔCp Syn_Hprt	ΔCp Syn_βact	ΔCp Hprt_βact	ΔCp Gapdh_βact		ΔCp Hprt_GAPDH	ΔCp Syn_GAPDH
37	16.8	16.9	20.7	20.9	16.4	16.4	15.4	15.4	-4.0	0.5	4.5	-1.1		5.6	1.6
	17		21.1		16.4		15.3								
38	21.1	21.1	24.5	25.0	20.0	20.0	19.3	19.1	-3.9	1.1	5.0	-0.9		5.9	2.0
	21		25.4		20.0		18.9								
39	21.2	21.3	24.3	25.1	21.2	20.9	19.7	19.5	-3.9	0.4	4.2	-1.4		5.6	1.8
	21.3		25.9		20.6		19.3								
40	17.6	17.6	21.2	21.2	17.2	17.4	16.0	16.1	-3.6	0.2	3.8	-1.4		5.2	1.6
	17.6		21.2		17.6		16.1								
41	18.4	18.5	23.3	22.9	18.9	19.0	16.9	16.9	-4.4	-0.5	3.9	-2.2		6.1	1.7
	18.6		22.5		19.1		16.8								
42	16	15.9	19.8	19.7	14.2	14.3	14.1	14.1	-3.8	1.6	5.4	-0.3		5.6	1.9
	15.8		19.5		14.4		14.0								
Average value:		18.5		22.5		18.0		16.8	-3.9	0.5	4.5	-1.2		5.6	1.7
Standard deviation (σ)		2.2		2.2				2.1							

**Supplement-Table 10:** Statistics significance levels of comparison groups based on the results in gene expression of syndecan-1 of aortic arch.

Syndecan-1 / Hprt	**C57 Black-WT**		**ApoE-/-**		Syndecan-1 / Gapdh	**C57Black-WT**		**ApoE-/-**	
Liver	**3m**	**8m**	**3m**	**8m**	Liver	**3m**	**8m**	**3m**	**8m**
C57Black-WT 3 м		p<	p<0.01		C57Black-WT 3 m		p<	p<0.05	
		0.001					0.001		
C57Black-WT 8 m	p<			p<	C57Black-WT 8 m	p<			p<
	0.001			0.001		0.001			0.001
АроЕ-/- 3 m	p<0.01			p<	АроЕ-/- 3 m	p<0.05			p<0.01
				0.001	АроЕ-/- 8 m		p<	p<0.01	
АроЕ-/- 8 m		p<	p<				0.001		
		0.001	0.001						

**Supplement-Table 11:** Statistics significance levels of comparison groups based on the results in gene expression of syndecan-1 of liver cells.

Syndecan-1 / Hprt	**C57Black-WT**		**ApoE-/-**		Syndecan-1 / Gapdh	**C57Black-WT**		**ApoE-/-**	
Liver	**3m**	**8m**	**3m**	**8m**	Liver	**3m**	**8m**	**3m**	**8m**
C57Black-WT 3 м					C57Black-WT 3 m			p<0.01	
C57Black-WT 8 m				p<0.001	C57Black-WT 8 m				p<
АроЕ-/- 3 m				p<0.05					0.001
АроЕ-/- 8 m		p<0.001	p<0.05		АроЕ-/- 3 m	p<0.01			p<0.05
					АроЕ-/- 8 m		p<	p<0.05	
							0.001		

**Supplement-Table 12:** ELISA data of ApoE-/- of 3-month-old mice.

**Nº of Mouse**	**Optical Density, 450 nm**	**Average of Optical Density, 450 nm**	**Concentration of Syndecan 1,**	Final Average Value, ng/ml * 20
			**pg/ml**	
1	0.385	0.405	1187.4	23.7
	0.424			
2	0.589	0.561	1836.2	36.7
	0.532			
3	0.422	0.448	1366.2	27.3
	0.473			
4	0.411	0.398	1158.3	23.2
	0.384			
5	0.456	0.446	1357.9	27.2
	0.435			
6	0.42	0.432	1301.7	26.0
	0.444			
7	0.426	0.445	1353.7	27.1
	0.463			
8	0.424	0.431	1297.6	26.0
	0.438			
9	0.462	0.471	1464.0	29.3
	0.48			
10	0.345	0.340	917.0	18.3
	0.334			
11	0.418	0.440	1335.0	26.7
	0.462			
12	0.386	0.389	1120.8	22.4
	0.391			

**Supplement-Table 13:** ELISA data of wild-type C57Black 8-month-old mice.

**Nº of Mouse**	**Optical Density, 450 nm**	**Average of Optical Density, 450 nm**	**Concentration of Syndecan 1, pg/ml**	Final Average Value, ng/ml * 20
13	0.438	0.431	1297.6	26.0
	0.424			
14	0.412	0.410	1208.2	24.2
	0.407			
15	0.359	0.382	1093.8	21.9
	0.405			
16	0.455	0.434	1308.0	26.2
	0.412			
17	0.421	0.429	1289.3	25.8
	0.437			
18	0.518	0.469	1455.6	29.1
	0.42			
19	0.455	0.471	1461.9	29.2
	0.486			
20	0.477	0.474	1474.4	29.5
	0.47			
21	0.337	0.387	1112.5	22.2
	0.436			
22	0.319	0.328	869.2	17.4
	0.337			
23	0.454	0.486	1526.3	30.5
	0.518			
24	0.586	0.526	1690.6	33.8
	0.465			

**Supplement-Table 14:** ELISA data of wild-type C57Black 3-month-old mice.

**Nº of Mouse**	**Optical Density, 450 nm**	**Average of Optical Density, 450 nm**	**Concentration of Syndecan 1, pg/ml**	Final Average Value, ng/ml * 20
25	0.258	0.261	590.5	11.8
	0.264			
26	0.229	0.234	476.1	9.5
	0.238			
27	0.326	0.330	877.5	17.5
	0.334			
28	0.341	0.351	962.8	19.3
	0.36			
29	0.266	0.258	578.0	11.6
	0.25			
30	0.31	0.301	754.8	15.1
	0.291			
31	0.356	0.359	998.1	20.0
	0.362			
32	0.352	0.338	908.7	18.2
	0.323			
33	0.295	0.286	692.4	13.8
	0.276			
34	0.369	0.364	1016.8	20.3
	0.358			
35	0.357	0.343	931.6	18.6
	0.329			
36	0.324	0.342	925.3	18.5
	0.359			

**Supplement-Table 15:** ELISA data of ApoE-/- of 8-month-old mice.

**Nº of Mouse**	**Optical Density, 450 nm**	**Average of Optical Density, 450 nm**	**Concentration of Syndecan 1, pg/ml**	Final Average Value, ng/ml * 20
37	0.415	0.410	1208.2	24.2
	0.404			
38	0.515	0.524	1684.4	33.7
	0.533			
39	0.623	0.615	2062.9	41.3
	0.607			
40	0.448	0.452	1382.9	27.7
	0.455			
41	0.533	0.534	1723.9	34.5
	0.534			
42	0.44	0.434	1308.0	26.2
	0.427			
43	0.522	0.535	1730.2	34.6
	0.548			
44	0.419	0.530	1707.3	34.1
	0.64			
45	0.52	0.529	1703.1	34.1
	0.537			
46	0.598	0.594	1975.6	39.5
	0.59			
47	0.576	0.559	1827.9	36.6
	0.541			
48	0.548	0.527	1694.8	33.9
	0.505			

**Supplement-Table 16:** Statistics significance levels of comparison groups based on the results of ELISA.

Soluble syndecan-1	**C57Black-WT**		**ApoE-/-**	
	**3m**	**8m**	**3m**	**8m**
C57Black WT 3 m		p<0.001	p<0.001	
C57Black WT 8 m	p<0.001			p<0.001
АроЕ-/- 3 m	p<0.001			p<0.001
АроЕ-/- 8 m		p<0.001	p<0.001	

**Supplement-Table 17:** Statistics significance levels of comparison groups based on the results of serum biochemistry.

Cholesterol	**C57Black-WT**		**ApoE-/-**	
	**3m**	**8m**	**3m**	**8m**
C57Black WT 3 m			p<0.001	
C57Black WT 8 m				p<0.001
АроЕ-/- 3 m	p<0.001			p<0.001
АроЕ-/- 8 m		p<0.001	p<0.001	

**Table 2:** Serum biochemistry parameters in mice relative to age and genotype. Retro-orbital collection method.

Plasma Analytes	C57Black 3m	C57Black 8m	ApoE^-/-^ 3m	ApoE^-/-^ 8 m
	Male (*n*=6)	Male (*n*=6)	Male (*n*=6)	Male (*n*=6)
Total cholesterol (mmol/l)	2.96±0.57	3.04±0.27	12.85±1.07	19.82±1.25
Triglycerides (mmol/l)	0.97±0.07	0.94±0.11	0.95±0.09	1.08±0.09
AST (u/1)	173.8±56.7	150.3±68.8	182.2±83.1	104.8±45.8
ALT (u/1)	65.0±11.4	63.8±27.0	72.6±19.7	43.1±15.9
Protein (g/l)	46.5±4.5	45.8±2.7	46.0±2.4	47.4±2.5
Albumin (g/l)	28.4±2.0	26.9±2.0	29.4±1.4	28.3±2.4
Creatinine (mmol/l)	34.0±5.0	26.9±2.0	27.0±5.0	29.4±5.0
Urea (mmol/l)	7.6±1.7	8.5±1.1	7.9±1.2	7.3±0.8
Ca (mmol/l)	2.21±0.17	2.29±0.45	2.19±0.18	2.1±0.14
Cl (mmol/l)	124.0±6.0	120.0±4.0	121.0±4.0	124.0±5.0
P (mmol/l)	3.08±0.5	3.26±0.37	2.96±0.55	2.99±0.47

## SUPPLEMENTARY MATERIAL

Supplementary material is available on the publishers website along with the published article.
